# From hepatic to hematopoietic: LRH‐1's expanding cellular repertoire to the immune system

**DOI:** 10.1111/febs.70589

**Published:** 2026-05-13

**Authors:** Lukas Meisinger, Alice Wiedmann, Rayan Hassan M. Elshikhidriss, Thomas Brunner

**Affiliations:** ^1^ Biochemical Pharmacology, Department of Biology University of Konstanz Constance Germany; ^2^ Department of Pharmacognosy, Faculty of Pharmacy University of Khartoum Sudan

**Keywords:** differentiation, granulocytes, inflammation, leukemia, macrophages, nuclear receptors, T lymphocytes

## Abstract

The nuclear receptor NR5A2 (Liver Receptor Homolog‐1, LRH‐1) has been well characterized in tissues of endodermal origin for the transcriptional control of development, metabolism, and steroidogenesis. In this minireview, we discuss the so far underappreciated expression and role of LRH‐1 in hematopoietic cells. We further highlight how deregulation of LRH‐1 may contribute to the pathogenesis of leukemia and immune cell‐mediated diseases, and how targeting LRH‐1 can be employed in immune cell‐targeted therapies. Given that LRH‐1 expression and function are highly tissue‐specific, we further discuss how these contextual differences may be exploited to achieve therapeutic selectivity, especially focusing on the myeloid and T cell lineage. Although current evidence for LRH‐1 functions in these immune cells is yet limited, its established role in the transcriptional regulation of development, differentiation, metabolism, proliferation, and cytokine expression of hematopoietic cells suggests a substantial and largely unexploited potential for therapeutic applications in leukemia and immunopathological diseases.

## Graphical abstract

The nuclear receptor LRH‐1 is a well‐characterized regulator of endodermal tissue. Yet, increasing evidence indicates that LRH‐1, although expressed at low levels, is also a critical regulator of the hematopoietic system. LRH‐1 regulates the immune system by contributing to immune cell‐specific functions. Consequently, its deregulation promotes the onset and progression of immune cell‐mediated diseases and inflammation and even hematopoietic cancer.

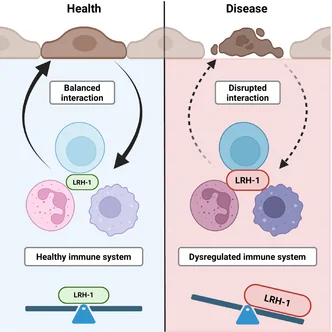

**Abbreviations**
15‐HETE: 15‐hydroxyeicosatetraenoic acid
AF: activation function
ALDH1B1: aldehyde dehydrogenase 1B1
ALL: Acute Lymphoblastic Leukemia
AP‐1: activator protein‐1
ATF4: activation transcription factor 4
C. albicans: Candida albicans
CBP: CREB‐binding protein
CCND1: cyclin D1
CCNE1: cyclin E1
CPT: carnitine palmitoyl transferase
CYP11A1: P450scc
CYP11B1: 11β‐hydroxylase
DAX1: dosage‐sensitive sex reversal
DBD: DNA‐binding domain
DLPC: dilauroyl phosphatidylcholine
FASLG: Fas (CD95) ligand
FGF7: Fibroblast Growth Factor 7
GDF15: Growth Differentiation Factor 15
GLS2: glutaminase 2
GLUT4: glucose transporter type 4
GR: glucocorticoid receptor (NR3C1)
HK2: hexokinase 2
IFN‐γ: interferon‐γ
IL: interleukin
LBD: ligand‐binding domain
LPS: lipopolysaccharide
LRH‐1: Liver Receptor Homolog‐1 (NR5A2)
MM: Multiple Myeloma
MYC: c‐Myc
NET: Neutrophil Extracellular Trap
NF‐κB: nuclear factor‐κB
NR: nuclear receptor
OCT4: octamer binding transcription factor 4
PBMC: peripheral blood mononuclear cells
PGC‐1α: peroxisome proliferator‐activated receptor gamma coactivator 1‐alpha
PPAR‐γ: peroxisome proliferator‐activated receptor‐γ
PROX1: prospero‐related homeobox 1
PTF1‐L: pancreas transcription factor 1
SF‐1: steroidogenic factor‐1 (NR5A1)
SHP: small heterodimer partner (NR0B2)
SMRT: silencing mediator for retinoic acid receptor and thyroid hormone receptor
SOD2: superoxide dismutase 2
SRC: steroid receptor coactivator
STARD: steroid acute regulatory protein
T1DM: Type 1 diabetes mellitus
T_C_
: cytotoxic T cell
T_H_
: T helper

## Introduction

The transcriptional regulation of gene expression and thus the availability of functional proteins is key to basically all cellular processes, and consequently the functionality of cells, tissue, and organs. Gene transcription is regulated by transcription factors, among which the nuclear receptor (NR) family of proteins represents the largest structurally related group [1]. NRs comprise a family of 49 receptors in mice and 48 receptors in humans. They are classified into 7 groups (NR0‐NR6) based on sequence similarity, ligand binding, and evolutionary relationships [1]. Alternatively, they are categorized into 4 functional groups (Type I‐Type IV) by their mode of action, respectively mode of regulation [2]. NRs are traditionally defined as ligand‐regulated, DNA‐binding transcription factors, tightly controlling cellular functions by regulating target gene expression. Yet, increasing evidence suggests also several non‐canonical functions of nuclear receptors, for example, as pioneer factors or mitotic bookmarks, therefore impacting the general transcriptional landscape [3, 4]. NRs have received extensive scientific and pharmaceutical attention, not only due to their key role in the regulation of numerous cellular processes, but also because they are “druggable” and their activity can be targeted by various pharmaceutical drugs, that is activators and inhibitors.

While a plethora of articles have reviewed the multifaceted role and regulation of individual NRs or NR groups, this present review aims to summarize the emerging role of the NR Liver Receptor Homolog‐1 (LRH‐1, NR5A2) specifically in hematopoietic cells, a combination which has been largely ignored thus far. LRH‐1 is a NR that is defined as a Type IV monomeric orphan receptor [5, 6]. It is expressed predominantly in tissues of endodermal origin and is known to be a master regulator of numerous cellular processes in these tissues and organs [6]. The crucial role of LRH‐1 in these processes is evidenced by the fact that mice with systemic deletion of the LRH‐1 gene are not viable and die *in utero* at day 6.5–7.5 of embryogenesis [7]. This role of LRH‐1 during development is likely related to the transcriptional control of the pluripotency factors Nanog (*NANOG*) and octamer binding transcription factor 4 (*OCT4*), thereby regulating stemness [8]. Additionally, LRH‐1 critically regulates cell proliferation via the transcriptional control of cyclin E1 (*CCNE1*) and D1 (*CCND1*), and c‐Myc (*MYC*), and thus contributes to tissue development, growth and renewal [9]. One of the extensively studied functions of LRH‐1 is its role as a regulator of cellular metabolism. Interestingly, LRH‐1 controls both, glycolysis as well as mitochondrial respiration. Thus, the glucose transporter type 4 (*GLUT4*), tissue‐specific hexokinases (*HK2, GCK*), glutaminase 2 (*GLS2*), and carnitine palmitoyl transferase (*CPT*) are transcriptional targets of LRH‐1, thereby boosting glycolysis as well as feeding the Krebs cycle by enhancing glutaminolysis and fatty acid oxidation [10, 11, 12]. LRH‐1 not only controls mitochondrial energy metabolism, but also processes that are critical for mitochondrial health. Thus, it was found to enhance superoxide dismutase 2 (*SOD2*) expression and NADPH production, thereby reducing the mitochondrial oxidative stress [11, 13]. Furthermore, LRH‐1 was shown to transcriptionally regulate the expression of the cholesterol transport protein steroid acute regulatory protein (*STARD1*), and the steroidogenic enzymes P450scc (*CYP11A1*) and 11β‐hydroxylase (*CYP11B1*) (Fig. 1A). These mitochondrial oxidoreductases are the rate‐limiting enzymes in steroidogenesis. Therefore, not surprisingly LRH‐1 is involved in the extra‐adrenal glucocorticoid synthesis in the intestine, which critically regulates intestine inflammation and anti‐tumor immune responses, as well as estrogen and progesterone synthesis in the ovaries [14, 15, 16].

**Fig. 1:**
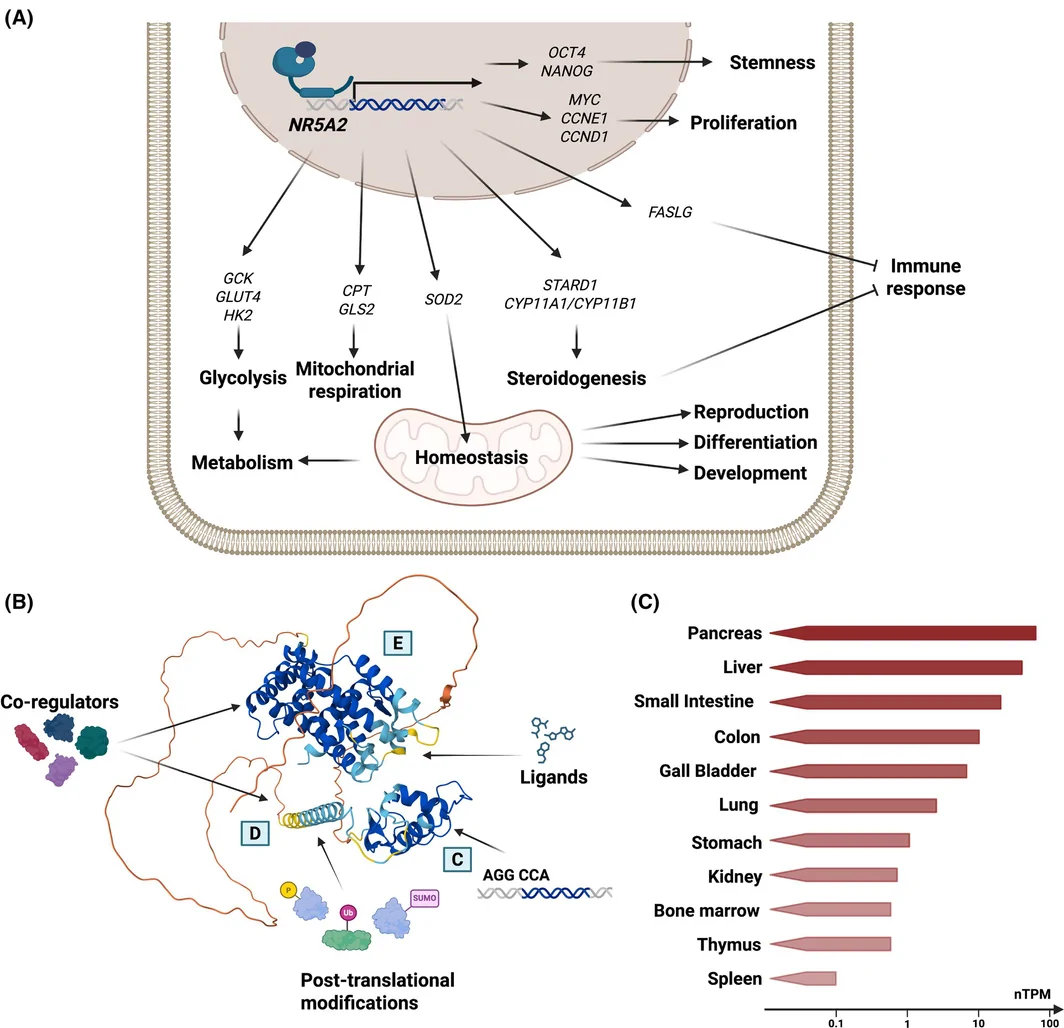
The tissue‐dependent expression levels, regulation and pleiotropic functions of LRH‐1. (A) Overview of processes that are transcriptionally regulated by NR5A2/LRH‐1. LRH‐1 regulates processes related to metabolism, mitochondrial function, reproduction, differentiation, development, proliferation, and stemness. Moreover, it influences the proximal tissue environment by the regulation of steroidogenesis and the TNF superfamily member Fas ligand, thus influencing the immune response. Glucokinase (GCK), glucose transporter type 4 (GLUT4), hexokinase‐2 (HK2), carnitine palmitoyltransferase (CPT), glutaminase 2 (GLS2), superoxide dismutase 2 (SOD2), steroidogenic acute regulatory protein (STARD1), cytochrome P450 11A1 (CYP11A1), cytochrome P450 11B1 (CYP11B1), Fas ligand (FASLG), c‐Myc (MYC), cyclin E1 (CCNE1), cyclin D1 (CCND1), octamer binding protein 4 (OCT4), Nanog (NANOG). (B) Structure of human LRH‐1 with suggested interaction sites for coregulators, ligands, and post‐translational modifications. The DNA‐binding domain (DBD) is indicated by C, the hinge region by D, and the ligand‐binding domain (LBD) by E. Predicted structure was generated using alpha fold (AF‐O00482‐F1‐v6) employing the LRH‐1 sequence (Uniprot: O00482). (C) Representation of relative expression levels, ordered by tissues, according to the human protein atlas (v25.0.proteinatlas.org). Expression levels are indicated by color gradient and scalebar. Created in https://BioRender.com.

Given the pleiotropic effects of LRH‐1 in a wide range of cellular processes, the tight control of its transcriptional activity appears critical. Like most NRs, LRH‐1 has four functional domains. Unlike most other NR, the N‐terminal (A/B) region does not contain a ligand‐independent activation function domain (AF‐1), known to interact with coactivators and co‐repressors. Especially, the most abundant isoform of LRH‐1 has only a shortened A/B domain due to alternative splicing. The DNA‐binding domain (DBD, C domain), which is highly preserved among different NRs, binds to the conserved hexameric half‐site motif AGG CCA in the promoter region of target genes [6]. However, the LRH‐1‐specific C‐terminal extension of the DNA‐binding groove additionally contains a minor groove extending its specificity to a YCA AGG YCR sequences (Y = pyrimidine, R = purine) [17, 18]. The hinge region (D domain) of LRH‐1 contains several amino acids that can be post‐translationally modified. While ERK1/2‐mediated phosphorylation of Serine 238 and 243 are associated with a transcriptional activation of LRH‐1, SUMOylation of Lysine 224 (human) and 289 (mouse) is associated with nuclear sequestration and transcriptional inhibition [19, 20, 21]. Furthermore, CUL4‐mediated ubiquitylation and degradation limits LRH‐1 protein levels and thus transcriptional activity [22]. The hinge region connects the DBD to the C‐terminal ligand‐binding domain (LBD, E domain). Together with the hinge domain, the LBD acts as an interaction platform for coregulators [17]. Co‐repressors, like small heterodimer partner (SHP, NR0B2), dosage‐sensitive sex reversal (DAX1, NR0B1), prospero‐related homeobox 1 (PROX1), silencing mediator for retinoic acid receptor and thyroid hormone receptor (SMRT), nuclear factor‐κB (NF‐κB), activator protein (AP‐1), and the glucocorticoid receptor (GRα, NR3C1), keep LRH‐1 in its transcriptionally inactive state, whereas binding of coactivators, such as steroid receptor coactivators (SRCs), CREB‐binding protein (CBP), peroxisome proliferator‐activated receptor gamma coactivator 1‐alpha (PGC‐1α) and β‐catenin, induces a conformational change and enhances LRH‐1 activity in a highly tissue‐specific manner [5, 23, 24, 25]. LRH‐1 is constitutively active, even in the absence of ligands. Due to the lack of identification of a *bona fide* endogenous ligand, LRH‐1 is still considered an orphan NR [5]. Nonetheless, X‐ray studies revealed binding of phospholipids to the LBD, and several reports described increased LRH‐1 activity in response to endogenous molecules (e.g., dilauroyl phosphatidylcholine, DLPC) and synthetic compounds (e.g., BL001 and RJW100) [26, 27, 28, 29, 30]. As discussed later, in particular synthetic agonists may have a therapeutic potential in various types of metabolic or inflammatory disorders. Similarly, LRH‐1 antagonists, like compound 3 and its more specific and potent derivate 3d2, or inverse agonists, like ML‐180 (SR1848), have been useful tools to study the role of LRH‐1 in different tissues and cells [31, 32] (Fig. 1B). In summary LRH‐1 is a ligand‐, coregulator‐ and post‐translation modification‐regulated transcription factor, controlling intra‐ and intercellular processes in a highly tissue‐specific manner.

## LRH‐1 expression and function

An interesting aspect of LRH‐1 is the relationship between expression levels and relative importance. As we will further discuss in this review, the absolute expression of LRH‐1 in a specific tissue does not necessarily predict the impact of tissue‐specific LRH‐1 deletion on the development and key functions of this tissue. LRH‐1 is predominantly expressed in organs and tissues of endodermal origin. Specifically, highest LRH‐1 expression is observed in the pancreas, followed by the liver and various parts of the gastrointestinal tract. Though substantially less abundant (often at 10 to 1′000‐fold lower levels), LRH‐1 is also detected in numerous other tissues, including the hematopoietic system (Fig. 1C), suggesting that LRH‐1 may have at the same time ubiquitous as well as tissue‐specific functions [33, 34].

### LRH‐1 in pancreas, liver, and intestine

In the pancreas, liver, and intestine, LRH‐1 has been extensively studied for its role in development and metabolism, but also for its contribution to tissue‐specific regulation of inflammation. The use of mice with organ‐ and tissue‐specific LRH‐1 deletion has been particularly useful in characterizing these functions. For example, mice with a hepatocyte‐specific LRH‐1 deletion develop a metabolic syndrome with increased hepatic lipid accumulation and type 2 diabetes, since LRH‐1 is critically involved in glucose and cholesterol metabolism and bile acid synthesis in the liver [35, 36]. In the exocrine pancreas, LRH‐1 cooperates with pancreas transcription factor 1 complex (PTF1‐L) to control the expression of digestive enzymes and their cholecystokinin‐induced release into the duodenum [37]. In the endocrine pancreas, LRH‐1 has been associated with β‐cell survival and insulin‐regulated glucose metabolism [27, 38]. In the intestinal epithelium, LRH‐1 is known to regulate stemness of crypt cells and proliferation of transit amplifying cells, and thereby epithelial cell renewal [9, 39]. Due to its role in the regulation of stem and progenitor cell proliferation, LRH‐1 has been implicated in tumor development of all of these three organs. Thus, LRH‐1 is also considered as an oncogene [34, 39].

Interestingly, LRH‐1 deficiency revealed also a connection to inflammation in these organs. Thus, LRH‐1 haploinsufficiency alters the transcription pattern in the pancreas, resulting in an AP‐1(Jun/Fos)‐dependent inflammation [24], whereas LRH‐1 activation can reduce pancreatic islet inflammation [27]. Similarly, hepatocyte‐specific LRH‐1 deletion is not only associated with lipid accumulation and steatosis but also liver inflammation [40] and resistance to TNF‐induced hepatitis [23]. A common underlying mechanism of LRH‐1 deficiency‐mediated pancreatic and hepatic inflammation is likely the heterodimerization of LRH‐1 with the transcription factors AP‐1 and NF‐κB and their reciprocal inhibition [23, 41]. Thus, LRH‐1 interacts with AP‐1, and reduced LRH‐1 levels in heterozygous mice likely lead to AP‐1 activation and associated inflammation in the pancreas [24]. Similarly, hepatic LRH‐1 deletion promotes spontaneous NFκB activation and NFκB‐driven inflammatory and anti‐apoptotic gene expression [23]. Furthermore, LRH‐1 may regulate inflammation in the liver also via the transcriptional control of aldehyde dehydrogenase 1B1 (*ALDH1B1*), which modulates reactive oxygen species levels and associated liver damage [40]. An example of even more direct LRH‐1‐regulated tissue inflammation is observed in the intestinal mucosa. Like its close homolog steroidogenic factor‐1 (SF‐1/NR5A1) in the adrenal cortex, LRH‐1 critically controls the expression of the steroidogenic enzymes *CYP11A1* and *CYP11B1* and thereby the synthesis of immunoregulatory glucocorticoids. Intestinal epithelial cell‐specific LRH‐1 deletion completely abolishes intestinal (local) glucocorticoid synthesis in response to immune cell activation, resulting in inflammatory bowel disease‐like inflammation [15, 16]. Although in this review we will primarily discuss a direct role of LRH‐1 in regulating immune cell functions, these examples already highlight the important role of LRH‐1 in controlling tissue‐specific inflammatory responses.

Despite the high abundance of LRH‐1 and the clearly important regulatory function LRH‐1 has in the transcriptional control of numerous processes in these tissues, it is noteworthy that LRH‐1 does not appear to be essential for the development of the liver and the intestine. Thus, hepatocyte‐specific deletion of LRH‐1 does still result in a normal sized liver, although associated with metabolic deficiencies and inflammation, and similarly LRH‐1 deficiency in intestinal epithelial cells leads to a mostly intact and functional epithelial layer [23, 42]. In marked contrast, induced genetic deletion of LRH‐1 in intestinal organoids results in rapid death of the epithelial cells [43]. This suggests that the lack of LRH‐1 during development and organogenesis of the liver and intestine can be largely compensated. As we will discuss later, even though LRH‐1 is barely expressed in immature and mature T cells, LRH‐1 deletion has a much stronger impact on T cell development and function.

### LRH‐1 expression in the hematopoietic system

As discussed above, LRH‐1 expression is particularly high in tissues and organs of endodermal origin, yet detectable in various other tissues. When directing our focus on the hematopoietic system, the first obvious observation is that LRH‐1 is indeed expressed in various types of hematopoietic cells, yet at very low levels. At the resting state, hematopoietic cells express LRH‐1 at about 10 to 1000‐fold lower levels than the pancreas or the liver [33, 34, 44, 45]. For this reason, a putative role of LRH‐1 in the regulation of hematopoietic cells had been largely ignored for a long time. Another interesting observation is the fact that terminally differentiated hematopoietic cells, like neutrophilic and basophilic granulocytes, express the highest levels of LRH‐1 in the hematopoietic system, at significantly higher levels than immune cells that still have proliferative potential, such as T cells [44, 45]. This suggests that LRH‐1 may have rather diverse functions in different hematopoietic cells, which go beyond its role in regulating cell cycle progression.

The first report of LRH‐1 expression in hematopoietic cells was rather indirect and derived from a study on the role of LRH‐1 in pancreatic tumors, where Benod and colleagues observed LRH‐1 immunoreactivity also in tumor‐infiltrating immune cells [46]. The functional significance of this accidental observation was, however, not further investigated at the time. Only approximately 10 years ago, specific studies investigating LRH‐1 expression and function in specific immune cell subsets had been initiated and revealed a surprisingly complex and distinct role of LRH‐1 in the regulation of various immune cell functions. These findings will be further and separately discussed for the myeloid lineage and T cell lineage.

### LRH‐1 in myeloid cells

The myeloid lineage of immune cells comprises dendritic cells, granulocytes, monocytes as well as macrophages. Most of these cell populations are involved in the innate immune response, yet also represent important links to the adaptive, specific immune response mediated by T and B cells, for example, by antigen presentation or source of modulatory cytokines. LRH‐1 is expressed in all myeloid cell populations, though only at low levels [44, 45].

Macrophages are highly adaptable immune cells that can change their behavior depending on signals from their environment [47]. Consequently, macrophages resident to different tissues express distinct markers and differ in their functional behavior. Traditionally, macrophages are grouped into two major functional states: classically activated (M1) macrophages, which promote strong inflammatory responses to eliminate pathogens, for example, by phagocytosis, and alternatively activated macrophages (M2), which are involved in inflammation resolution, tissue repair, and immune regulation. While M1 macrophages can be induced upon stimulation with bacterial products, such as lipopolysaccharide (LPS), or interferon‐γ (IFN‐γ), M2 macrophage differentiation is triggered by the cytokines interleukin (IL)‐4 and IL‐13 and involves transcriptional reprogramming by the transcription factors peroxisome proliferator‐activated receptor‐γ (PPAR‐γ) and STAT6 [47, 48].

LRH‐1 was first described in macrophages in the context of M2 differentiation and effector functions. The NR PPAR‐γ is activated by lipophilic ligands, such as 15‐hydroxyeicosatetraenoic acid (15‐HETE). 15‐HETE is generated from arachidonic acid in a 5‐lipoxigenase‐ or *CYP1A1/CYP1B1*‐dependent manner. Interestingly, like many other cytochrome C P450 enzymes, also *CYP1A1* and *CYP1B1* are transcriptional targets of LRH‐1. IL‐13, which drives M2 macrophage differentiation, induces LRH‐1 expression in macrophages in a STAT6‐dependent manner. Via the transcriptional regulation of CYP1A1 and CYP1B1, LRH‐1 is thus an important link between IL‐13 signaling and 15‐HETE/PPAR‐γ‐mediated differentiation and reprogramming of M2 macrophages. M2 macrophages are known to be critical for the innate immune response against the pathogenic yeast *Candida albicans* (*C. albicans*). IL‐13‐induced and LRH‐1/PPAR‐γ‐regulated expression of mannose receptor (CD206), recognizing mannose‐rich structures of *C. albicans*, and Dectin‐1, as well as inducing the production of reactive oxygen species appears to be critically involved in the resolution of *C. albicans* infections. Consequently, mice with macrophage‐specific LRH‐1 deletion show reduced *C. albicans* clearance and overall survival, which can be restored upon administration of 15‐HETE [49, 50].

This interesting study not only describes for the first time a critical role of LRH‐1 in immune cells, but suggests a specific regulatory role of LRH‐1 in distinct macrophage populations. However, the overall role of LRH‐1 in macrophages appears to be far more complex and to extend to other macrophage populations. M1 macrophages produce the pro‐inflammatory cytokines TNF, IL‐6, and IL‐1β upon LPS stimulation. Pharmacological inhibition of LRH‐1 or downregulation by RNA interference significantly inhibits the LPS‐induced expression and release of these pro‐inflammatory cytokines. On the other hand, deletion of SHP, one of the endogenous co‐repressors of LRH‐1, results in increased LPS‐induced TNF release due to increased LRH‐1 activity [51]. Excitingly and further discussed in the context of LRH‐1‐based therapeutic approaches, *in vivo* administration of the pharmacological LRH‐1 inhibitor 3d2 significantly improved LPS/GalN‐induced liver damage in a macrophage/TNF‐dependent murine model of hepatitis. Thus, while LRH‐1 appears to have a general role in the regulation of macrophage differentiation and function, specific regulatory processes seem to be macrophage subset‐specific (Fig. 2).

**Fig. 2:**
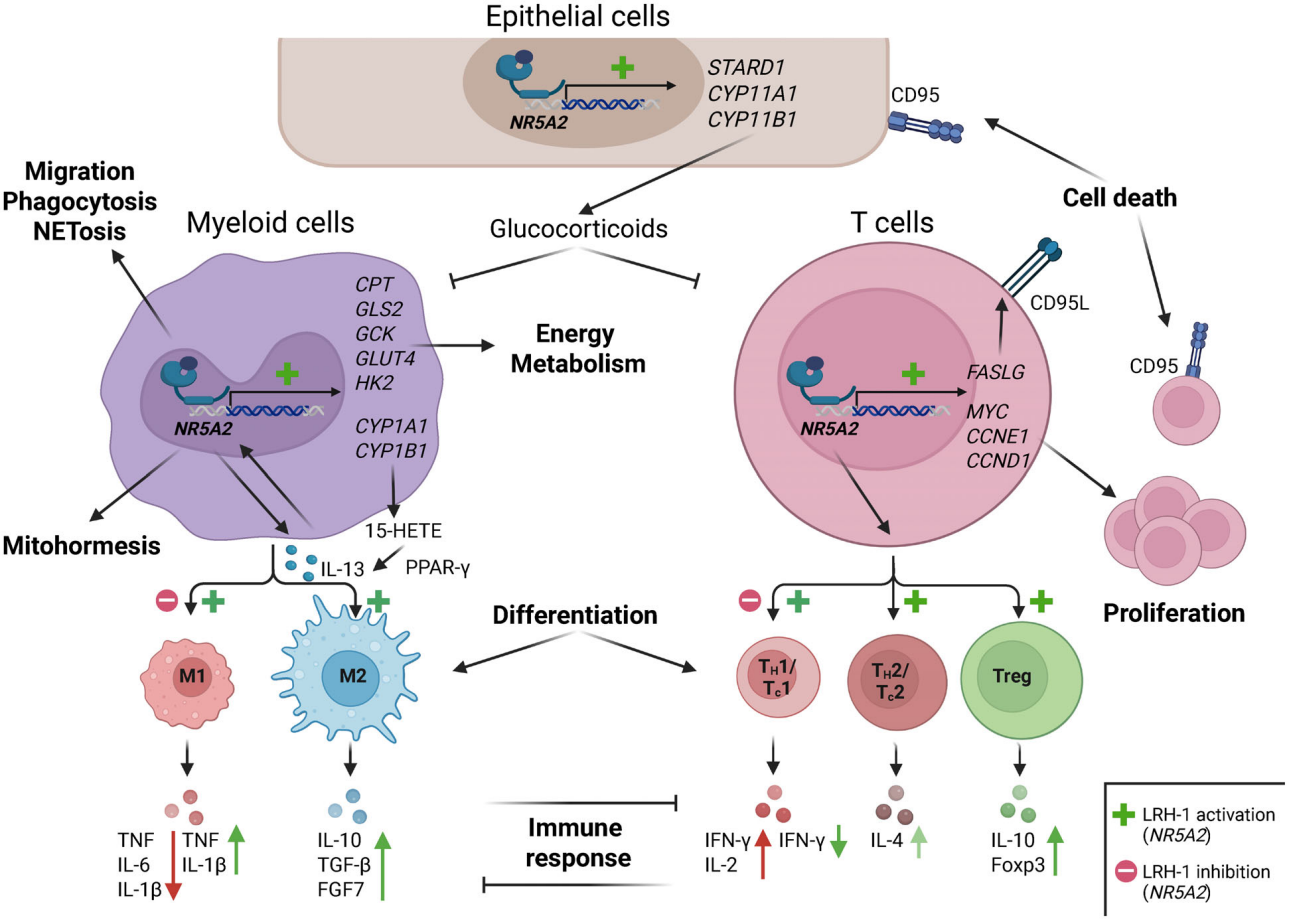
LRH‐1, a central regulator of myeloid and T cell effector functions. LRH‐1 (NR5A2) transcriptionally activates the cholesterol transport protein steroid acute regulatory protein (STARD1) and the steroidogenic enzymes cytochrome P450 (CYP) CYP11A1 and CYP11B1 in epithelial cells, thereby contributing to extra‐adrenal glucocorticoid synthesis suppressing myeloid and T cell activation. In myeloid cells, activation or inhibition of LRH‐1 modulates neutrophil migration, phagocytosis, and Neutrophil Extracellular Trap formation (NETosis), as well as mitohormesis in macrophages. LRH‐1 further controls cellular energy metabolism by inducing the expression of carnitine palmitoyltransferase (CPT), glutaminase 2 (GLS2), glucokinase (GCK), glucose transporter 4 (GLUT4), and hexokinase 2 (HK2). LRH‐1‐dependent expression of CYP1A1 and CYP1B1 promotes the production of the peroxisome proliferator‐activated receptor gamma (PPAR‐γ) ligand 15‐hydroxyeicosatetraenoic acid (15‐HETE), which, among other things, drives anti‐inflammatory (M2) macrophage differentiation in an interleukin (IL)‐13‐dependent manner. Furthermore, NR5A2 expression is induced by IL‐13. Accordingly, modulation of LRH‐1 activity shifts the balance between pro‐ and anti‐inflammatory myeloid cell states, including pro‐inflammatory M1 versus M2 macrophages, and differentiation of regulatory T cells (Treg). LRH‐1 activation induces the expression of anti‐inflammatory cytokines, including IL‐10, transforming growth factor (TGF‐β), and fibroblast growth factor (FGF7) in M2 macrophages, as well as IL‐10 and forkhead box P3 (Foxp3) in T cells. LRH‐1 inhibition reduces TNF, IL‐6, and IL‐1β expression in macrophages, while increasing IFN‐γ and IL‐2 secretion in T cells. In contrast, LRH‐1 activation enhances TNF and IL‐1β responses in M1 macrophages, suppresses IFN‐γ in T_H_1/T_C_1 cells and slightly promotes IL‐4 in T_H_2/T_C_2. LRH‐1 also regulates activation‐induced proliferation by increasing cell cycle‐associated genes, including c‐Myc (MYC), cyclin E1 (CCNE1), and cyclin D1 (CCND1). Additionally, LRH‐1 induces Fas ligand (CD95L, FASLG) expression, contributing to activation‐induced cell death of T cells and promoting apoptosis of epithelial cells. Red arrows and minus symbols indicate effects of LRH‐1 inhibition, green arrows and plus symbols indicate effects of LRH‐1 activation, and faint green arrows indicate mild effects of LRH‐1 activation. Created in https://BioRender.com.

Interestingly, these effects of LRH‐1 inhibition in M1 macrophages are likely closely related to impaired energy metabolism by glycolysis and glutaminolysis [51]. While to some degree a simplification, it is generally accepted that M1 macrophages preferentially use glycolysis for ATP production, whereas M2 macrophages rely more on oxidative phosphorylation. Furthermore, M1 macrophages appear to have an impaired Krebs cycle, resulting in the accumulation of microbicidal and pro‐inflammatory metabolites [52]. Of interest, LRH‐1 transcriptionally regulates *GCK* and *GLS2* expression, thereby directly impacting glycolysis and the Krebs cycle. Thus, it is feasible that the role of LRH‐1 in M1 macrophages and the release of pro‐inflammatory cytokines is mostly related to the regulation of energy metabolism, whereas in M2 macrophages it involves direct transcriptional control of M2 differentiation.

These studies suggest rather fixed and defined roles of LRH‐1 in differentiated macrophage populations. However, much like polarized macrophage populations maintain a certain plasticity upon transcriptional reprograming, also the role of LRH‐1 and the impact of LRH‐1 inhibition, respectively activation, on macrophages appears to be highly context‐dependent. Type 1 diabetes mellitus (T1DM) is a devastating autoimmune disease resulting in the destruction of the insulin‐producing β‐cells in the pancreatic islets. Though the disease is strongly mediated by auto‐aggressive T cells, there is a relevant contribution by inflammatory innate immune cells, including macrophages. While it was previously found that LRH‐1 directly regulates β‐cell survival and that the small molecular LRH‐1 agonist BL001 can improve experimental T1DM in murine models [27, 38], a recent study by Cobo‐Vuilleumier *et al*. indicates that the beneficial effect of LRH‐1 activation may also be by reprograming pro‐inflammatory M1 macrophages to an anti‐inflammatory, tolerogenic phenotype [53, 54]. Activation of LRH‐1 in macrophages by BL001 was associated with reduced secretion of pro‐inflammatory cytokines, yet increased expression of the immunoregulatory cytokines IL‐10 and TGF‐β, M2 macrophage‐promoting IL‐4 and Fibroblast Growth Factor 7 (FGF7), driving a tolerogenic and reparative macrophage phenotype (Fig. 2). Interestingly, similar anti‐inflammatory effects of LRH‐1 stimulation were also observed in dendritic cells, indicating that LRH‐1's immunoregulatory role is not limited to macrophages [53].

As discussed above, LRH‐1 has an important role in the regulation of the cellular energy metabolism via the transcriptional control of *GCK* and *GLS2*. At the same time, LRH‐1 activation appears to suppress the expression of mitochondrial proteins in M1 macrophages [53]. In line with this observation, stimulation of M1 macrophages with BL001 resulted in increased glycolysis and reduced mitochondrial oxidative phosphorylation. Excessive mitochondrial respiratory activity is often associated with increased reactive oxygen species generation and mitochondrial damage [55]. It is thus interesting that LRH‐1 activation appears to support mitophagy and thus the removal of damaged mitochondria, as well as the induction of mitohormesis [53] (Fig. 2), a mitochondria‐nucleus crosstalk, ultimately limiting pro‐inflammatory responses [56, 57, 58]. In this context, it has been found that LRH‐1 activation by BL001 does not restore oxidative metabolism in pro‐inflammatory macrophages; rather, it induces a mitohormetic response through adaptive mitochondrial signaling as evidenced by the reduction of basal mitochondrial respiration capacity upon LRH‐1 activation without impairing maximal respiration, demonstrating preserved mitochondrial competence despite reduced baseline activity. LRH‐1 activation seems to result in a pro‐inflammatory paralyzed state of macrophages through triggering of mitohormesis as indicated by increased expression and protein level of activation transcription factor 4 (ATF4), the principal driver of mitohormesis and its downstream target Growth Differentiation Factor 15 (GDF15) along with decreased expression levels of IL‐1β and the inflammasome sensor NLRP3 [53].

Despite the fact that LRH‐1 exerts obviously contradictory effects on inflammatory responses in macrophages, these discrepancies can be harmonized by considering the cellular context and timing of LRH‐1 activation and action. In healthy monocytes, LRH‐1 activity supports early inflammatory signaling; thus, its inhibition attenuates the LPS‐induced initiation of pro‐inflammatory cascades [51]. In contrast, macrophages in an autoimmune or diseased context, like T1DM, are already metabolically reprogrammed towards an inflammatory phenotype. Under these conditions, LRH‐1 activation likely promotes mitochondrial restoration and immunometabolic reprogramming, which enables transformation from a persistent inflammatory to a more tolerogenic phenotype [53].

Beyond these regulatory roles of LRH‐1 in macrophages and dendritic cells, recent work has expanded LRH‐1's immunoregulatory functions to neutrophils and their role in the tumor microenvironment. Neutrophilic granulocytes, together with basophilic granulocytes, are among the highest LRH‐1‐expressing hematopoietic cells [44, 45]. Since neutrophils are frequently present in solid tumors as well as their metastatic lesions and required to support T cell responses, modulation of their activity could be crucial for the efficacy of cancer treatment [59, 60]. LRH‐1 has been found to influence migration, phagocytosis, and NETosis of neutrophils (the induced release of Neutrophil Extracellular Traps, containing mitochondrial DNA and anti‐microbial proteins) (Fig. 2), and their interactions with T cells. Thus, pharmacological inhibition of LRH‐1 inhibits phagocytosis of bacteria and tumor cells, yet promotes NETosis of neutrophils, suggesting that LRH‐1 differentially regulates specific neutrophil effector pathways. Along with a regulatory role of neutrophils in tumor growth, pharmacological inhibition of LRH‐1 accelerated growth of transplanted mammary tumors, whereas stimulation with BL001 resulted in reduced tumor growth [60]. Although the pharmacological effects of LRH‐1 antagonists and agonists may not be solely related to direct effects of neutrophils, this relevant *in vivo* study further extends the focus of LRH‐1 to neutrophilic granulocytes and their role in anti‐tumor immune responses.

Although in immune cells LRH‐1 appears to be highest expressed in basophilic granulocytes [44, 45], its role in basophils is currently completely unexplored. To date, no studies have specifically addressed the functions of LRH‐1 in these rare types of granulocytes. Although very rare (approx. 0.5% of human peripheral blood leukocytes), basophilic granulocytes are a rich source of immunoregulatory cytokines, such as IL‐4 and IL‐13 [61, 62], and are increasingly being recognized as critical regulators of type 2 inflammatory processes, such as allergies and asthma. In analogy to the role of LRH‐1 in M2 macrophage differentiation, it is tempting to speculate that LRH‐1 may have a similar role in the differentiation and effector functions of basophils. Indeed, ongoing unpublished research in our group indicates that genetic deletion as well as pharmacological inhibition of LRH‐1 in murine basophils affects their differentiation and activation‐induced cytokine production (R. Elshikhidriss and T. Brunner, unpublished).

### LRH‐1 in T cells

The preceding chapter summarized the current knowledge on the role of LRH‐1 in cells of the innate immune system, although only limited information is available on granulocytes, which exhibit the highest *NR5A2* expression in the hematopoietic lineage. The investigations on LRH‐1 in cells of the adaptive immune system, that is, lymphoid lineage, have also only been initiated recently and are still relatively sparse. Up to now, LRH‐1 expression has been confirmed in B lymphocytes [44, 45], yet its role in different B cell subsets is completely lacking. In marked contrast, genetic and pharmacological studies clearly indicate a critical role of LRH‐1 in the regulation of T cell development and various T cell effector functions.

The co‐receptor CD4 is a marker of T helper and regulatory T cells. It first becomes expressed during T cell development during the so‐called double‐positive (CD4^+^ CD8^+^) state of thymocytes [63]. Since double‐positive thymocytes can develop into both mature CD4^+^ as well as CD8^+^ T cells, CD4 promoter‐based Cre expression and floxed target gene deletion is being frequently used to study genes in immature and mature T cells [64]. LRH‐1 expression is being detected in T lymphocytes throughout their different developmental stages [33, 65]. Furthermore, mitogenic activation of mature T cells further induces its expression. Interestingly, CD4 promoter‐Cre‐mediated LRH‐1 gene deletion has only a very minor impact on the size of the thymus, and the number and relative distribution of various thymocyte subsets [65]. While at first glance this may suggest that LRH‐1 is not essential for thymic development, this observation may primarily be related to the timing of LRH‐1 deletion during thymic development, since deletion at earlier developmental stages has drastic impacts on thymocyte numbers and development (A. Wiedmann, T. Brunner, unpublished). In marked contrast to the thymus, peripheral T cells in mice with CD4‐Cre‐mediated LRH‐1 deletion are severely affected. Spleen size and weight, and total T cell numbers are drastically reduced and mice are lymphopenic. Specifically, CD4^+^ T cells are reduced by two thirds, and CD8^+^ T cells by 50%, while B cells remain at normal levels and numbers. When analyzing the functional consequences of LRH‐1 deletion, it is noted that LRH‐1 deficiency does not affect the expression of early T cell activation markers (CD69, CD25), but does severely impair T cell receptor‐induced proliferation, which is also being confirmed *in vivo* and by pharmacological LRH‐1 inhibition *ex vivo* [65]. Very much like in macrophages, the effects of LRH‐1 inhibition and activation appear not to be necessarily reciprocal, since *ex vivo* activation of LRH‐1 in human peripheral blood mononuclear cells (PBMCs) from T1DM patients also results in diminished T cell proliferation [27, 53]. Given that PBMCs contain also myeloid cells, this inhibitory effect may at least in part also be related to effects of LRH‐1 activation in accessory immune cells. Interestingly, the proliferative defect appears to be subset‐specific. CD4^+^ T cells are more affected by LRH‐1 deficiency than CD8^+^ T cells. Thus, in a T cell transfer model of colitis, LRH‐1‐deficient CD4^+^ T cells completely fail to expand *in vivo* and to induce intestinal inflammation. Similarly, transferred LRH‐1‐deficient CD4^+^ regulatory T cells were unable to suppress intestinal inflammation by LRH‐1‐proficient disease‐promoting CD4^+^ T cells. Thus, LRH‐1 deficiency affects both effector T helper cells as well as regulatory T cells. In contrast, during lymphocytic choriomeningitis virus infection, LRH‐1‐deficient cytotoxic T cells expand normally, yet fail to control viral spreading [65]. Likely, this immunodeficiency is either related to reduced cytotoxic activity or lack of T helper cell support. The mechanisms underlying the LRH‐1‐regulated proliferation of T cells involve interactions with proteins of the WNT/β‐catenin pathway, and associated transcriptional induction of cell cycle‐regulating genes, such as *MYC*, *CCNE1*, and *CCND1* [9, 25, 65, 66]. Interestingly, the LRH‐1 target gene *MYC* not only drives proliferation of immature and mature T cells [65, 67, 68], but also orchestrates metabolic reprogramming upon T cell activation and during T cell development [68], further supporting the above‐discussed putative role of LRH‐1 in the regulation of immune cell metabolism.

Beyond LRH‐1's role in T cell proliferation and potentially metabolism, LRH‐1 has been implicated in shaping the cytokine profile and subset polarization of mature T cells. LRH‐1 deficiency drastically enhances T helper (T_H_) 1/cytotoxic T cell (T_C_) 1‐associated cytokine secretion, including IFN‐γ and IL‐2, whereas LRH‐1 activation slightly favors T_H_2/T_C_2 responses with mildly increased IL‐4 production and reduced IFN‐γ^+^ CD8^+^ T cells [53, 65]. The observed T_H_1/T_C_1 shift in the absence of LRH‐1, together with the mildly enhanced T_H_2/T_C_2 polarization upon LRH‐1 activation, suggests a role for LRH‐1 in promoting T_H_2/T_C_2 polarization, while restraining T_H_1/T_C_1 polarization (Fig. 2). It is still unclear whether LRH‐1 directly or indirectly regulates master T cell polarization genes, such as *GATA3*, supporting T_H_2/T_C_2 differentiation [69], or *TBX21* (Tbet), which drives T_H_1/T_C_1 differentiation [70]. Nevertheless, modulating LRH‐1 activity or expression of LRH‐1 in T cells severely impacts T cell differentiation and cytokine secretion, and thus shows clearly some analogy to the role of LRH‐1 in M1/M2 macrophage differentiation.

The critical role of LRH‐1 in regulating T cell differentiation and effector functions suggests that LRH‐1‐based therapies could be successfully applied to various types of immunopathologies. The beneficial effects of LRH‐1 inhibitors on macrophage‐mediated hepatitis [51] and LRH‐1 ligands on T1DM [27, 53] have already been discussed above. In T cells, LRH‐1 not only regulates activation‐induced proliferation, but also the expression of the apoptosis‐inducing Fas ligand (*FASLG*, CD95L) [33]. CD95L is involved in T cell homeostasis by downsizing T cell numbers at the end of an immune response [71, 72]. In addition, CD95L‐mediated killing of tissue cells is also an underlying reason for T cell‐mediated pathologies, as for example hepatitis [73]. Investigating the regulation of CD95L transcription, we identified two functional LRH‐1 binding sites in the promoter of the *FASLG* gene. Consequently, pharmacological inhibition of LRH‐1 significantly impairs activation‐induced CD95L expression and associated apoptosis induction. Excitingly, *in vivo* administration of the LRH‐1 inhibitor 3d2 effectively inhibited CD95L expression and hepatocyte apoptosis induction in a murine model of T cell‐mediated hepatitis, while having only minimal direct effects on hepatocytes with very high LRH‐1 expression [33].

### LRH‐1 in hematopoietic tumors

The important role of LRH‐1 in the regulation of stemness, metabolism, and proliferation already strongly suggests that LRH‐1 may have oncogenic potential. Thus, not surprising, LRH‐1 deregulation is observed in a variety of solid tumors, in particular tumors derived from tissues with high LRH‐1 expression. These include colorectal cancer, pancreatic tumors, hepatocellular carcinomas, as well as breast cancer. Increased LRH‐1 expression in tumors from these endodermal tissues has been linked to increased proliferation, resistance to apoptosis, enhanced metastatic potential, and altered drug metabolism [34, 66]. In colorectal cancer, LRH‐1 activity has also been associated with tumor‐autonomous synthesis of immunoregulatory glucocorticoids, and the escape from surveillance and destruction by the immune system [16]. LRH‐1 deletion, downregulation, or pharmacological inhibition in tumor organoids or tumor cell lines from these tissues promotes cell cycle arrest and cell death [66, 74, 75, 76]. While the oncogenic potential of LRH‐1 in these tumors is well established, and pharmacological inhibition of LRH‐1 demonstrates therapeutic effects in proof‐of‐concept studies, nearly nothing is known so far on the role of LRH‐1 in hematopoietic tumors. LRH‐1 is detected in a large number of leukemia, lymphoma, and myeloma cell lines; however, its role in the regulation of hematopoietic tumors is thus far nearly unexplored [77, 78]. Yet, limited functional insight is available for Multiple Myeloma (MM) and Acute Lymphoblastic Leukemia (ALL). In a recent study, Li *et al*. demonstrated decreased overall and progression‐free survival of MM patients with elevated LRH‐1 expression [79]. In line with this observation, unpublished analysis of the TARGET gene data set by our group also indicates an increased mortality in ALL patients with high LRH‐1 expression levels (L. Meisinger, T. Brunner, unpublished). Mechanistically, LRH‐1 appears to support tumor development by promoting the expression of cell cycle‐regulating proteins, such as cyclin D1/E1 and c‐Myc [25, 79]. Furthermore, LRH‐1 is enhancing tumor cell survival, and its pharmacological inhibition directly promotes BIM‐dependent apoptosis [25]. Synthetic glucocorticoids, such as dexamethasone, are a standard therapy in the treatment of MM and ALL patients. Though very effective, many patients develop resistance to glucocorticoids [80]. LRH‐1 is also involved in this resistance to glucocorticoid‐induced apoptosis. Downregulation or pharmacological inhibition of LRH‐1 strongly sensitizes MM and ALL cell lines to dexamethasone. Interestingly, this resistance appears to be tightly linked to the ability of LRH‐1 to heterodimerize with other transcription factors and NRs [25, 79]. We and others have previously shown that LRH‐1 interacts with AP‐1 and NF‐κB, resulting in the mutual inhibition of these transcription factors [23, 41]. As a consequence, tissue‐specific deletion of LRH‐1 promotes spontaneous NF‐κB activation and associated inflammation, as seen in the liver [23]. Similarly, heterodimerization is also involved in the glucocorticoid resistance of T‐ALL cells [25]. We have seen that upon glucocorticoid‐induced translocation, the glucocorticoid receptor is captured in the nucleus by LRH‐1, resulting in the transcriptional inactivation of both NRs. Thus, LRH‐1 is a co‐repressor of the glucocorticoid receptor and *vice versa*. Consequently, downregulation or pharmacological inhibition of LRH‐1 liberates the glucocorticoid receptor, reestablishes its activity, and thereby promotes BIM expression and associated intrinsic apoptosis [25]. Excitingly, the sensitizing effect of LRH‐1 inhibition on glucocorticoid‐induced T‐ALL cell death is observed also *in vivo* in primary patient‐derived T‐ALL cells transplanted into immunodeficient mice (PDX), while having no obvious side effects on normal tissues. This further supports the notion that LRH‐1‐based therapies could be successfully applied in tumor therapy.

Also, in hematopoietic tumors, LRH‐1 regulates the energy metabolism. In T‐ALL cells LRH‐1 downregulation results in enhanced phosphorylation of AMPK, indicating reduced ATP production and energy stress, while in MM cell lines LRH‐1 activity is associated with the regulation of the mitochondrial respiratory chain and oxidative phosphorylation [25, 79]. Interestingly, an additional mechanism of cell death regulation may apply to MM cells. In the past, LRH‐1 had been implicated in the transcriptional regulation of lipid metabolism [36]. Likewise, in MM LRH‐1 may regulate cellular phospholipid remodeling, thereby modulating membrane lipid composition and sensitivity to ferroptosis induction [79]. However, it is currently unclear whether this alternative mechanism of cell death regulation applies also to other hematopoietic tumors.

In summary, though yet limited, these studies suggest an interesting and so far neglected role of LRH‐1 in hematopoietic tumors, with great therapeutic potential to overcome basal or acquired drug resistances.

## LRH‐1—a therapeutic target in inflammatory diseases and hematopoietic tumors?

As discussed in previous chapters, LRH‐1‐modulating substances, including LRH‐1 agonists and inhibitors, have been very useful to interrogate and dissect various LRH‐1‐regulated processes in different cell types and tissues. Pharmacological inhibitors have been especially suitable since deletion of the LRH‐1 gene is often not tolerated by many target cells with a high addiction to LRH‐1 (e.g., tumor cell lines), and RNA interference‐based downregulation offers a limited observation window due to metabolic shutdown, energy crisis, and reduced proliferation and survival of targeted cells [25]. In contrast, LRH‐1 inhibitors, such as ML‐180 (SR1848) [32] and compound 3d2 [31], can be administered to target cells shortly before investigating LRH‐1‐regulated processes. Furthermore, even though LRH‐1 has an important role in various physiological processes in different organs and tissues, LRH‐1 inhibitors can be applied also *in vivo*, and single doses or repetitive administration do not seem to critically affect the physiological functions of organs with high LRH‐1 expression, such as the liver and the intestine. Nonetheless, therapeutic doses applied *in vivo* were able to reduce growth of transplanted tumors, or reduce inflammatory processes and associated tissue destruction [25, 33, 51]. This interesting “therapeutic window” may be also affected by large differences in LRH‐1 expression in healthy tissue and target cells, such as hematopoietic tumors or various immune cells. Thus, it is feasible that a given dose of LRH‐1 inhibitors may inhibit only insignificant amounts of LRH‐1 in tissues, such as the liver and intestine, and therefore have only minimal side effects. In contrast, the relatively low levels of cellular LRH‐1 in hematopoietic target cells may be more readily saturated by inhibitors, ultimately having a more pronounced effect on hematopoietic target cells. Thus, pharmacological LRH‐1 inhibition may become an especially interesting approach in the treatment of hematological tumors or immunopathologies due to the relatively low levels of LRH‐1 expression in target cells, but high dependency on LRH‐1‐regulated processes. Nonetheless, the spectrum, specificity and activity of currently available LRH‐1 inhibitors is rather limited, and further improvements in inhibitor development will be necessary. LRH‐1 (NR5A2) is very homologous to SF‐1 (NR5A1), and both NRs bind to the same consensus sequences in the promoter of target genes [17, 30, 81]. Similarly, some ligands as well as inhibitors may bind to the LBD of both NRs, and thereby not only affect LRH‐1‐regulated processes [82, 83]. Although some drug screens for LRH‐1 inhibitors took into consideration potential inhibition of SF‐1, potential interference with other NRs, including SF‐1, will be an issue in the development of specific LRH‐1 inhibitors. Similarly, improved activities of LRH‐1 inhibitors will be required to target LRH‐1‐expressing cells *in vivo* more efficiently. A better understanding of the molecular interactions of inhibitors, respectively ligands, with various parts of the ligand‐binding pocket, and the related consequences for protein conformation and recruitment of co‐repressors may contribute to further improving pharmacological LRH‐1 inhibitors.

While for the treatment of hematopoietic tumors pharmacological LRH‐1 inhibitors may be of particular interest, various preclinical models of autoimmune, inflammatory and metabolic diseases also highlighted the potentially beneficial effects of LRH‐1 activation by synthetic ligands. For example, mice with T1DM seem to greatly benefit from treatment with the synthetic LRH‐1 ligand BL001 due to improved pancreatic β‐cell survival and reduced immune cell activation [27, 53]. Furthermore, combining synthetic ligands with deep pocket interaction (e.g., RJW100) [83, 84] with phospholipid moieties, preferentially anchoring the ligand in the mouth of the ligand‐binding pocket, results in significantly improved LRH‐stimulating activity. Such hybrid molecules, that is, phospholipid mimetics, are 10CA [85] and 6 N [86], which show greatly improved activities and have been successfully tested in preclinical mouse models of intestinal inflammation [43]. Also, the identification of natural ligands may be of considerable therapeutic interest. Though *bona fide* endogenous LRH‐1 ligands have not been identified yet, various phospholipids, such as phosphatidylethanolamine, phosphatidylglycerol and phosphatidylcholine, are known to alter LRH‐1 activity [29, 87, 88, 89]. Especially DLPC, though rarely detected *in vivo*, has been successfully used to trigger LRH‐1‐regulated processes *in vivo*, and thereby improve experimental liver steatosis and parenteral nutrition‐associated choleostasis [90]. Given that the LRH‐1 ligand‐binding pocket can accommodate structurally very diverse lipophilic substances [91], even screening of natural plant‐ and bacteria‐based compounds for LRH‐1‐stimulating activity may hold great promises in the development of LRH‐1‐targeting drugs.

Taken together, despite its low expression, LRH‐1 is a NR with diverse and important physiological and pathophysiological functions in different immune cells. Even though the role of LRH‐1 in distinct immune cell populations and the regulation of their effector functions is barely investigated, recent studies demonstrate that a deregulation of LRH‐1 activities in hematopoietic cells contributes to the development of immunopathologies and hematological cancer. Therefore, modulating LRH‐1 activity through specific agonists and antagonists represents a promising therapeutic strategy for the treatment of immunopathologies or blood cancer.

## Autor contributions

All authors contributed to the conceptualization and writing of the review.

## Conflict of interest

The authors declare no conflict of interest.
